# *QuickStats*: Percentage* of Women Aged 25–44 Years Who Had Ever Used Infertility Services,^†^ by Type of Service — National Survey of Family Growth, United States, 2006–2010 and 2015–2019

**DOI:** 10.15585/mmwr.mm7040a5

**Published:** 2021-10-08

**Figure:**
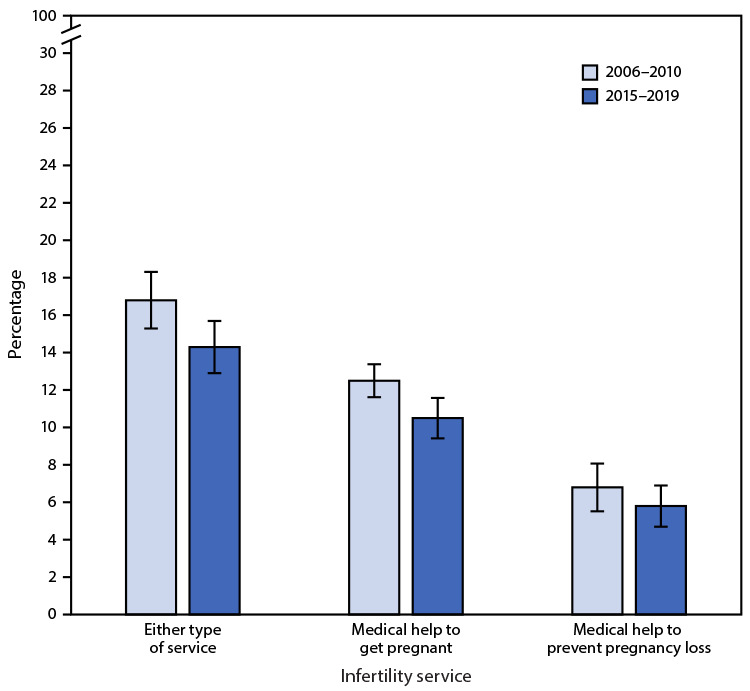
During 2015–2019, among women aged 25–44 years, 14.3% had ever used any infertility services, down from 16.8% during 2006–2010. The percentage who had ever used medical help to get pregnant declined from 12.5% during 2006–2010 to 10.5% during 2015–2019, but the difference in the percentage ever using medical help to prevent pregnancy loss (6.8% during 2006–2010 and 5.8% during 2015–2019) was not statistically significant. During both periods, a higher percentage had ever received medical help to get pregnant than had ever received medical help to prevent pregnancy loss.

**Source:** National Survey of Family Growth, 2006–2010 and 2015–2019. https://www.cdc.gov/nchs/nsfg/index.htm

